# Isolated Penile Fracture and Complete Urethral Injury After a Motorcycle Accident: A Case Report and Literature Review

**DOI:** 10.7759/cureus.55535

**Published:** 2024-03-04

**Authors:** Hussein Gossadi, Hisham Abu Eishah, Ali Autwdi, Mohammed Abualgasem

**Affiliations:** 1 Department of Urology, Sabia General Hospital, Jazan, SAU

**Keywords:** urology, trauma, tri-tubular, urethral injury, penile injury

## Abstract

Tri-tubular penile fracture (PF) is a rare urological subdivision of PFs commonly caused by a blow to the erect penis during sexual intercourse or aggressive manipulation. PF associated with complete urethral injury and bleeding is an extremely rare presentation.

This is a case report of a healthy 20-year-old male who presented to the emergency room after a motorcycle accident, experiencing rapid penile swelling and urethral bleeding. The accident happened while he was riding his motorcycle with a full erection. The patient reported a tearing sensation, immediate detumescence, and excruciating penile pain. A clinical diagnosis of PF was made, and the patient was immediately taken to the operating room for surgical intervention. At the three-month follow-up, the patient reported satisfactory erections and good voiding function. This case highlights the importance of immediate surgical intervention and urethral evaluation to avoid PF complications.

## Introduction

Penile fracture (PF) is defined as the traumatic tearing of the tunica albuginea of the tumescent penis, resulting in the rupture of one or both of the corpora cavernosa [1]. PF mainly occurs when unphysiological bending forces are applied to an erect penis during sexual intercourse or due to aggressive penile manipulation. Patients typically report hearing a cracking or clicking sound followed by instantaneous detumescence and pain [2].

PF is a rare urological emergency that must always be treated immediately. Between 9% and 20% of PF cases also present with urethral injury, particularly in patients with urinary symptoms, such as urine retention and urethral bleeding [3]. The incidence of associated urethral injury is significantly higher (20%-25%) in the Western world compared to Asian countries (3%-6%) [4]. We report a rare urological case of complete urethral disruption with rupture of both corpora cavernosa. A literature review regarding PF is also included.

## Case presentation

We report the case of a 20-year-old healthy male who arrived at the emergency room after a motorcycle accident, experiencing rapid penile swelling and urethral bleeding over the previous five hours. The accident happened while he was riding his motorcycle in a rural area. He was steering the motorcycle with one hand and holding his phone with the other and had a full erection. He lost his balance and fell on his abdomen after driving over a small dirt bump. The patient reported a tearing sensation, immediate detumescence, excruciating penile pain, and frank bleeding from the urethra, followed by penile swelling. The pain was exacerbated by the inability to pass urine. The patient was not taking any medication and did not have any allergies. His medical history showed no penile or urological congenital deformities or injuries. Written consent to report this case was obtained from the patient.

Physical examination revealed normal vital signs with a soft and nontender abdomen, although the bladder was palpable. Penoscrotal examination showed a flaccid, circumcised penis with significant swelling and ecchymosis (*eggplant* appearance), and there was blood spotted on the urethral meatus. The scrotal and perineum examinations were unremarkable. A clinical diagnosis of PF concomitant with urethral injury was made, and the patient was immediately taken to the emergency operation room for surgical exploration due to the emergency status of the case presentation.

**Figure 1:**
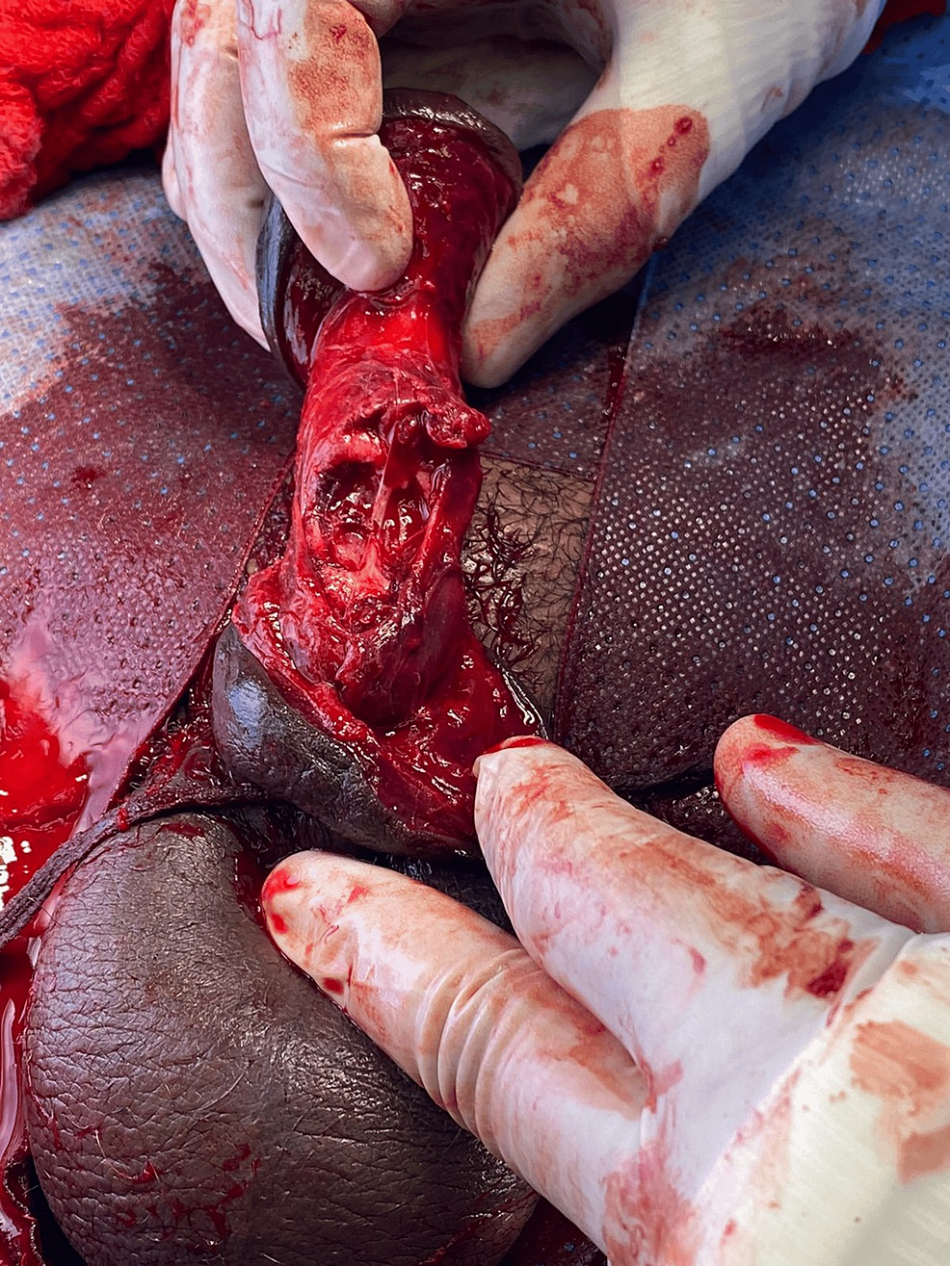
Intraoperative photo after hematoma evacuation demonstrating the bilateral transverse rupture of the corpora cavernosa.

**Figure 2:**
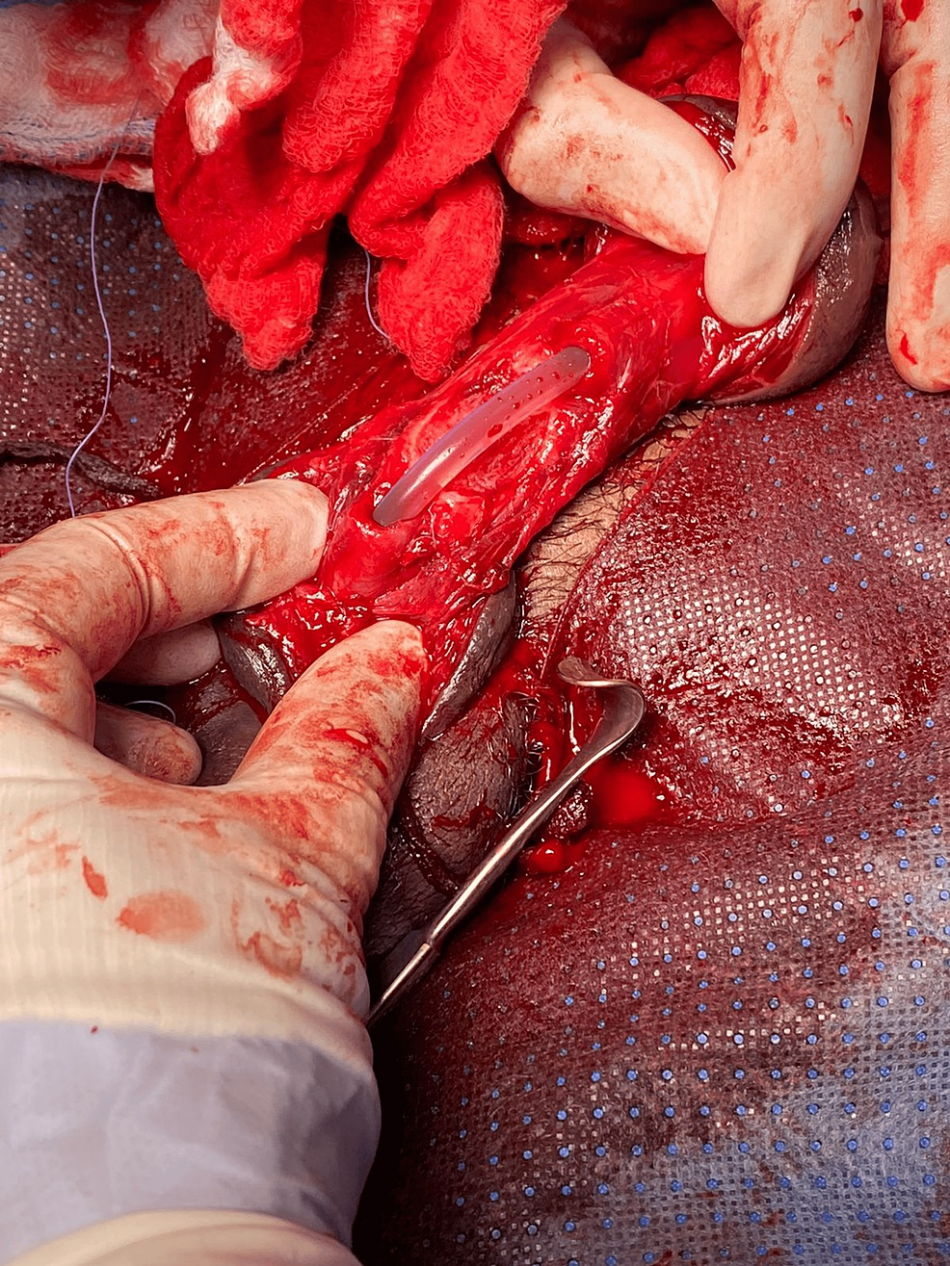
Intraoperative finding of a complete urethral rupture.

**Figure 3:**
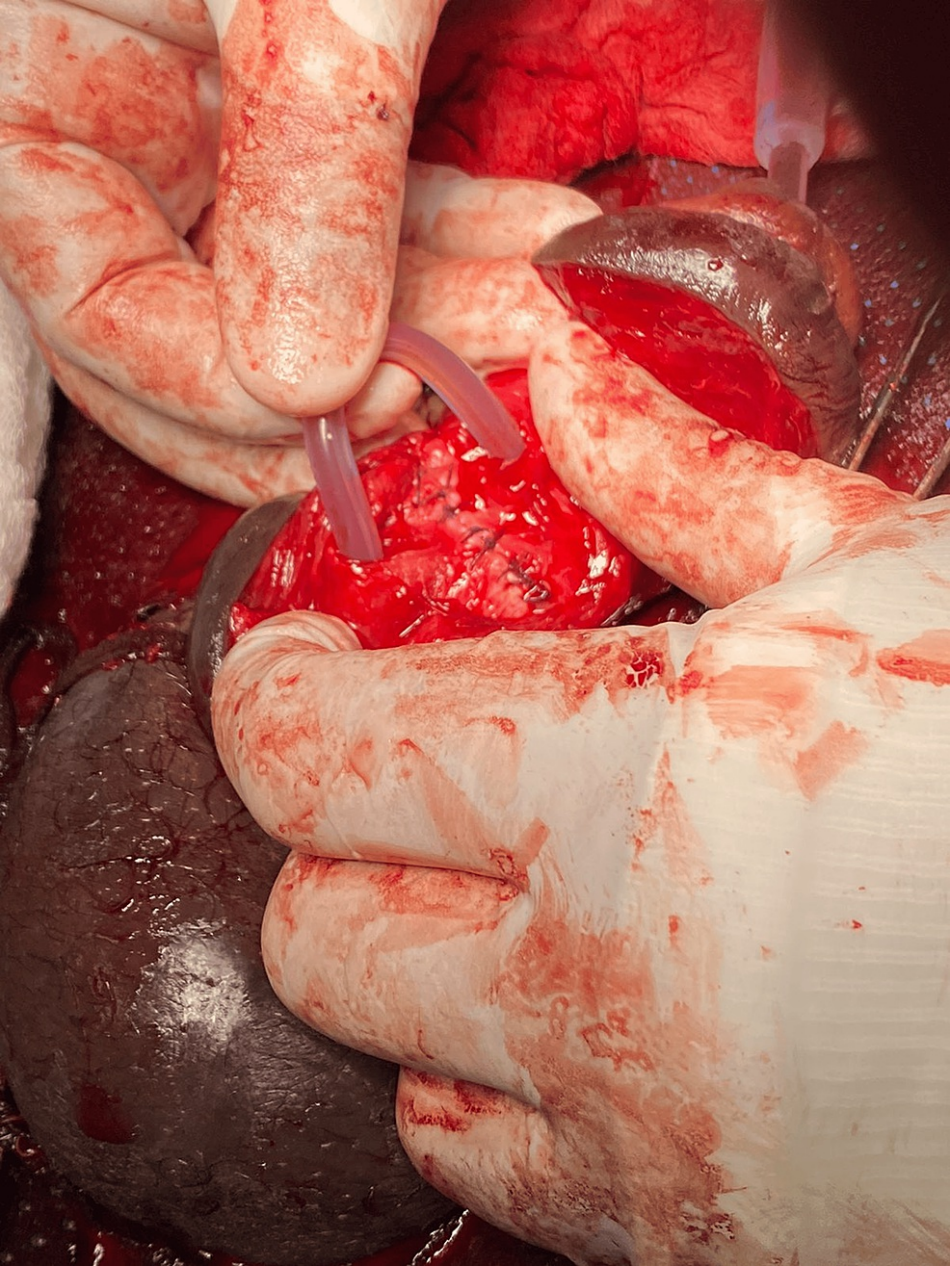
Suture of both corpus cavernosum tears and insertion of an 18-French silicone catheter.

On the theater table, fluoroscopy was out of service, so we could not do a retrograde urethrogram to see the urethral status. Flexible urethroscopy was used for visual examination of the urethral mucosa, which showed a blind end as a result of a large clot and a hidden proximal urethral end. A circumferential sub-coronal incision and penile degloving were performed. Hematoma evacuation was carried out, and an obvious disruption of Buck’s fascia was found during the exploration. Transverse traumatic transection of the corpora cavernosa was observed bilaterally (Figure 1). Additionally, complete urethral transection was noted at the level of the mid-penile shaft (Figure 2). The defects were repaired using a simple interrupted technique (Figure 3).

Exploration of the corpus spongiosum revealed a complete urethral disruption, which was repaired using an 18-French silicone catheter with parachute-interrupted stitching. The stitching was performed end-to-end, mucosa to mucosa, ensuring a tension-free anastomosis. An artificial erection and leak test were conducted, demonstrating a watertight closure of the corpora. Irrigation was applied to the wound, and the surgical incision was closed in layers. A broad-spectrum antibiotic was given during the patient’s hospital stay.

The patient recovered well, and he was discharged on postoperative day two with antibiotics and an 18-French Foley catheter. He was also advised to abstain from any sexual stimulation. During the first postoperative review in the clinic on day five, the wound was found to be healthy, but the patient mentioned recurrent painful nocturnal erections that were mitigated by pseudoephedrine. Two weeks later, the catheter was removed and the patient passed urine freely under uroflowmetry (maximum flow rate was 12.6 mL/s; total volume was 363.5 mL; Table 1).

**Table 1:** Uroflowmetry.

Uroflowmetry	
Average flow rate	4.8 mL/s
Maximum flow rate	12.6 mL/s
Flow at 2 seconds	3.8 mL/s
Acceleration	0.9 mL/s^2^
Time to max flow	0:13.3 minute:s
Total volume	363.3 mL

**Figure 4:**
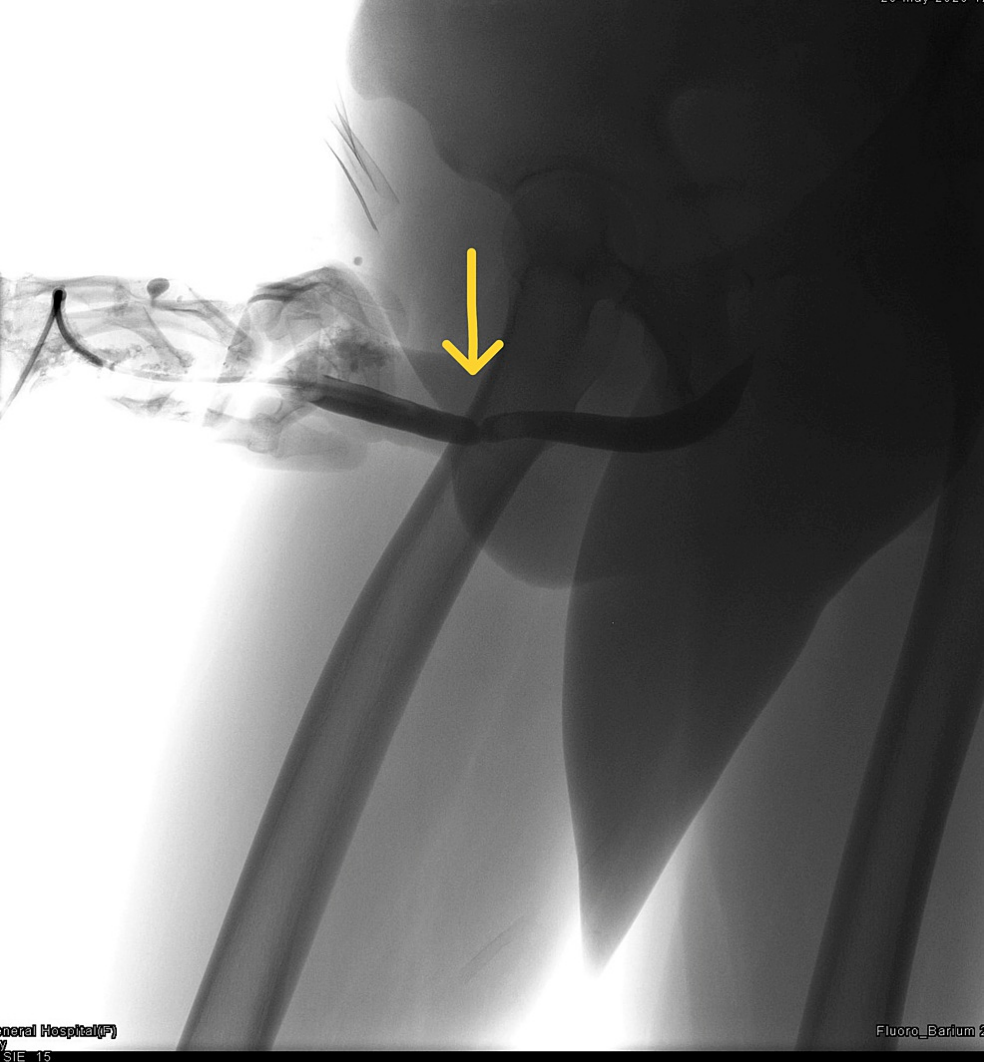
Retrograde urethrogram showing an anterior urethral circumferential stricture.

Three months later, the patient underwent a retrograde urethrogram, which demonstrated anterior urethral circumferential stricture with no extravasation contrast (Figure 4). The patient mentioned good voiding and painless erections and resumed normal life without physical or psychological issues.

## Discussion

The fracture of the penis is a rare urological emergency that occurs around once in every 175,000 emergency cases. It is defined as a traumatic rupture of the tunica albuginea of the corpora cavernosum that mainly occurs during intercourse when the penis strikes the perineum or due to aggressive manipulation, e.g., masturbation [5]. Other causes include rolling over on one’s penis when sleeping and externally directed blunt trauma [6]. Another etiology of PF that has been cited in the literature is taghaandan, which is a self-inflicted injury that involves the forced bending of an erect penis to achieve rapid detumescence, gunshot wounds, and industrial accidents [7-9].

A PF that is caused by sexual athleticism or simple manipulation is generally regarded as blunt trauma. The most common cause of this type of fracture is vigorous vaginal intercourse and bending during intercourse, something that occurs more often in the Western world [10]. In contrast, literature on Middle Eastern and Gulf countries states that forced penile manipulations, such as kneading the penis to achieve rapid detumescence and masturbation, are the most common causes of PF, accounting for 65% of cases [11].‏

Clinically, the patient hears a cracking sound, accompanied by intense penile pain and swelling. Other commonly associated symptoms are immediate penile detumescence, bruising, shape deformity, and contralateral deviation to the lesion [12]. Urethral meatus bleeding and acute urine retention or voiding-related symptoms may indicate a possible urethral-associated injury. However, the absence of these symptoms does not rule out the possibility of a urethral injury. In most patients, Buck’s facia remains maintained, which means the hematoma that develops only affects the penis [13].

In 10% to 20% of cases, the injury may expand to affect the corpus spongiosum and the urethra [14]. The incidence of urethral injuries occurring with PF is between 0% and 3% in Asia and the Middle East and between 20% and 38% in Europe and the United States. Urethral injuries tend to be partial [15-16], and complete urethral disruption, such as in the current case, is extremely uncommon.

An appropriate medical history and a thorough physical examination lead to a diagnosis in most cases, and further investigations are not usually needed. In unclear cases, additional imaging such as penile ultrasonography can support the diagnosis, but operator-dependent methods have significant false-negative rates. Magnetic resonance imaging (MRI) offers significantly better soft tissue image quality and can detect tunical tears noninvasively [17-19].

Early reports recommended conservative management, such as cold compressions, elastic bandages, analgesia, anti-inflammatories, antibiotics, and erection suppression medications; however, long-term follow-ups showed that patients often developed complications like penile curvature, pain, erectile dysfunction, and arteriovenous fistulas [20]. Conservative management is now being replaced with immediate surgical interventions for patients who likely have PF to avoid later complications [21]. Patients who are managed surgically may have a slight penile curvature but report no loss of sexual function [18].

In general, both conservative and surgical management of PF can lead to complications, some of which have devastating physiological and psychological consequences. However, cases managed conservatively have significantly higher incidences of complications. Early postoperative period complications include penile hematoma, wound infection, penile skin necrosis, penile abscess, and psychiatric disturbance [22-24]. Late complications of surgical management include erectile dysfunction, induration, penile aneurysm, and penile curvature [25]. We recommend urgent surgical intervention for all patients with a high likelihood of PF. Emergency surgical repair is the best option for a favorable functional prognosis and makes a full recovery possible [26-27].

## Conclusions

Penile fracture accompanied by complete urethral injury is extremely rare. Clinical diagnosis is sufficient in most cases, but additional investigations such as ultrasound sonography and MRI can help confirm the fracture. Immediate surgical exploration and repair should be performed to reduce the possibility of serious and long-term complications. An associated urethral injury commonly involves a bilateral cavernosal rupture, as seen in the current case, for which immediate surgical repair is mandatory.
